# Acid-resistant *Bacillus velezensis* effectively controls pathogenic *Colletotrichum capsici* and improves plant health through metabolic interactions

**DOI:** 10.1128/aem.00340-25

**Published:** 2025-06-23

**Authors:** Yuxiang Peng, Chi Zhou, Fangying Qiu, Di Peng, Xinyu Wang, Xin Li

**Affiliations:** 1 Longping Branch, College of Biology, Hunan University 214170, Changsha, China; 2 Hunan Institute of Microbiology 639864, Changsha, China; 3 Hunan Engineering Research Center on Excavation and Utilization of the Endophytic Microbial Resources of Plants, Changsha, China; 4 College of Environment and Ecology, Hunan Agricultural University 662258, Changsha, China; The University of Tennessee Knoxville, Knoxville, Tennessee, USA

**Keywords:** *Bacillus velezensis*, *Colletotrichum capsici*, acid-resistant acclimated, metabolic pathway

## Abstract

*Colletotrichum capsici* is the etiological agent of Capsicum anthracnose. *Bacillus velezensis* has traditionally been recognized as an effective biocontrol agent; however, its efficacy decreases due to soil acidification. In this study, we domesticated *Bacillus velezensis* XY40-1 along an acid resistance gradient, resulting in a strain capable of growth at pH 4, and might adapt to acidic environments by regulating genes related to spore formation. Notably, the domesticated *Bacillus velezensis* XY40-1 exhibits significant antagonistic activity against *Colletotrichum capsici* in acidic dual cultures and effectively reduces the disease index in *Capsicum*. The domesticated strain employs a direct antifungal strategy under acidic conditions, with the production of amylocyclicin, regulated by *acnA*, potentially serving as a primary mechanism through which *Bacillus velezensis* combats *Colletotrichum capsici*. Conversely, under neutral conditions, domesticated *Bacillus velezensis* focuses on bolstering its defense mechanisms by increasing the expression of *katA*, *ahpF*, and *ahpC* genes to detoxify peroxides. In addition, a dual RNA-Seq analysis comprehensively investigated the acid tolerance mechanisms and defensive responses of *B. velezensis* and the pathogenic mechanisms of *C. capsici*, providing a foundation for the practical application of *B. velezensis* as a biocontrol agent. These findings offer important insights into the impact of soil acidification on plant disease suppression and contribute to the development of sustainable agricultural practices.

**IMPORTANCE:**

Recently, the increasing issue of soil acidification has worsened anthracnose disease in Capsicum, caused by *Colletotrichum capsici*. Our study demonstrated that *Bacillus velezensis* can effectively inhibit the growth of *Colletotrichum capsici*. However, the molecular mechanisms underlying the interaction between *Bacillus velezensis* and *Colletotrichum capsici* remain largely unexplored. Here, we developed an interaction system between *Bacillus velezensis* and *Colletotrichum capsici* to explore their dynamic relationship. By employing dual RNA-Seq methods, we were able to comprehensively investigate the acid tolerance mechanisms and defense responses of *Bacillus velezensis*, alongside the pathogenic mechanisms of *Colletotrichum capsici*. This establishes the groundwork for utilizing *Bacillus velezensis* as an effective biocontrol agent in agriculture.

## INTRODUCTION

*Capsicum* is one of the most economically significant crops in the world, with the global cultivation of peppers exceeding 3.68 Mha (1). *Colletotrichum capsici* (*C. capsici*) is a major disease affecting capsicum. To effectively manage capsicum anthracnose, which poses a substantial threat to the quality, yield, and economic value of capsicum, it is crucial to address this issue (2). Recent research indicates that biological control methods offer several advantages, including being non-toxic to humans and animals, environmentally friendly, and not inducing resistance (3). As a result, biocontrol has gradually emerged as a prominent alternative to chemical interventions in plant disease management strategies.

In recent years, *Bacilli* have emerged as innovative biocontrol microorganisms, demonstrating their efficacy through various mechanisms. These microorganisms synthesize antimicrobial substances that effectively inhibit the growth of plant pathogens (4). Furthermore, the metabolites produced by Bacilli activate pathways associated with induced systemic resistance (ISR) in plants, thereby protecting against pathogenic microbial attacks (5). *Bacillus velezensis* is a well-established biocontrol bacterium recognized for its antagonistic properties against a range of pathogens (6). This bacterium has shown effectiveness as a biocontrol agent in the management of apple bitter rot and anthracnose. They mainly inhibit spore germination and hyphal growth by secreting secondary metabolites that degrade their cell walls (7). Previous studies have predominantly focused on the transcriptomes of individual bacterial or fungal species (8, 9). Only a limited number of investigations have successfully characterized the transcriptional changes that occur during the interaction between biocontrol bacteria and plant pathogens through the application of a dual RNA-seq approach (10). For example, *Bacillus velezensis* and molecules derived from it have been shown to inhibit the growth of *F. graminearum in vitro* and limit FHB disease progression *in planta* when applied as biocontrol agents (11). However, the interaction between *B. velezensis* and *C. capsici* has been notably underexplored. Specifically, the differential expression of genes associated with *B. velezensis* and *C. capsici*, as well as the intricate interplay between these genes, remains largely ambiguous.

Acid deposition and irrational fertilization practices are well-documented factors that can significantly alter the physicochemical properties of soil (12). These changes often lead to a range of detrimental effects, including soil acidification, the accumulation of pathogenic bacteria, and a reduction in beneficial microorganisms (13). As soil acidification intensifies, it creates an unfavorable environment for the growth of *B. velezensis*, thereby diminishing its efficacy against *C. capsici* and exacerbating the prevalence of this pathogen. However, there is currently a paucity of research dedicated to enhancing the tolerance of *B. velezensis* to acidic conditions and elucidating the mechanisms underlying the interaction between acid-resistant *B. velezensis* and *C. capsici*, particularly with respect to potential transcriptional changes. By improving the tolerance of *B. velezensis* to acidic soil environments and augmenting its resistance to *C. capsici* under these conditions, this research aims to elucidate the biological control mechanisms employed by *B. velezensis* against *C. capsici*. Ultimately, this study seeks to provide a viable strategy for managing *C. capsici* and to establish a foundation for the development and application of *B. velezensis* as an effective biocontrol agent.

## MATERIALS AND METHODS

### Biological materials, culture conditions, and pH adjustment

Cultures of *C. capsici* were obtained from the Hunan Vegetable Research Institute. *B. velezensis* XY40-1 was isolated from the leaves of *Capsicum* and has been preserved by our research group. *C. capsici* was cultivated on potato dextrose agar (PDA) at a temperature of 28˚C, while *B. velezensis* XY40-1 was grown on Luria-Bertani agar (LBA) under the same temperature conditions.

LB Miller liquid medium: 10 g/L Tryptone, 5 g/L yeast extract, 10 g/L NaCl, and pH 7.0. PDA (Pyeast) medium was prepared according to the directions of the manufacturer at 46 g/L, pH 6.0. 1 mol/L HCl and NaOH were used to adjust the pH. The medium was then autoclaved at 121°C for 15 min. Potassium phosphate buffer (PPB, g/L): 12.52 g/L KH_2_PO_4_, 1.39 g/L K_2_HPO_4_, and pH 2.5.

The initial pH of the sterile soil was 6.0. Over a period of 90 days, the soil was watered every 5 days with solutions of pH 4 and pH 7. The acidic solution was adjusted using HCl, while the alkaline solution was adjusted using quicklime (CaO). Soil pH was measured in a 1:2.5 (vol/vol) soil/water ratio using an FE 20 pH meter (Mettler-Toledo International Inc., China) after 30 d and 90 d (Table S1).

### Acid resistance and growth characteristics of *B. velezensis*

To carry out the acid-resistant acclimation of XY40-1, the pH of the medium was gradually decreased. LB Miller liquid medium was used with an initial pH of 7, as a control. The pH values of the LB Miller liquid medium were adjusted to 5.0, 4.5, 4.0, 3.5, 3.0, 2.5, and 2.0. Using pH 5 as a starting point for domestication, each pH was subjected to five passages, with repeated domestication to ensure complete adaptation. The viable bacteria count was recorded to assess genetic stability. After observing satisfactory growth, the domesticated bacterial solution was evenly spread on the corresponding acidic plate. Subsequently, it was inoculated into the medium of the next lower pH, continuing this process sequentially.

Fresh single colonies of *B. velezensis* were selected under different pH treatments before and after acclimation. These colonies were inoculated into 50 mL of acclimated liquid medium and shaken at 37℃ and 190 rpm for 24 h. The viable bacteria were subsequently observed using an optical microscope and counted using the blood cell counting plate method. Different seed solutions were then adjusted to ensure a uniform concentration of viable bacteria. The resulting bacterial solution was inoculated into 50 mL of acclimated liquid medium at a 1% inoculation rate. The culture was shaken at 37°C and 190 rpm, with samples taken every 3 h to measure absorbance at a wavelength of 600 nm.

### Antibacterial activity of *B. velezensis in vitro* and *in vivo*

To compare the antibacterial activity of acclimated and unacclimated *B. velezensis* against pathogens at different pH, a dual culture method was employed *in vitro*. Initially, *C. capsici* were inoculated at the center of PDA medium with pH 4 and pH 7. Subsequently, acclimated and unacclimated *B. velezensis* were separately inoculated 2 cm away from the pathogen center in the medium using a cross-inoculation method. Meanwhile, the control group was not subjected to *B. velezensis* inoculation. Concurrently, acclimated *B. velezensis* and unacclimated *B. velezensis* were cultured in solid LB medium at pH 4 and pH 7, respectively, and then incubated at 28℃ for 5–7 days. Following incubation, the colony diameter was measured, and the antibacterial rate was determined. Each group consisted of six replicates.

The antibacterial rate (%) was calculated using the following formula: antibacterial rate (%) = (colony diameter of control group − colony diameter of treatment group)/colony diameter of control group × 100.

In the *in vivo* experiment, the roots of pepper plants were irrigated with both acclimated and unacclimated *B. velezensis* at a concentration of 1 × 10⁸ CFU/mL in soils with pH 4 and 7. 48 hours post-irrigation, a solution containing *C. capsici* spores at a concentration of 1 × 10⁶ CFU/mL was directly applied to the soil. Sterile water served as a blank control, while plants inoculated solely with the pathogen spore solution acted as a negative control. The plants were then incubated for 7 days, during which disease indices were photographed and recorded to assess disease severity. Each treatment was repeated at least three times.

The disease index (%) was calculated using the following formula: disease index (%) = [100 × ∑ (number of diseased plants at each level × representative value of that level)]/(total number of plants × representative value of the highest level).

### Sample preparation, RNA extraction, and sequencing

In the preceding experiments, interaction systems between *B. velezensis* and *C. capsici* were established. After 10 days of co-culture, both acclimated and unacclimated *B. velezensis* interacting with *C. capsici* at pH 4 and pH 7 (designated X4_X, X7_X, WX4_X, and WX7_X) were collected in the study. The acclimated *B. velezensis* (designated CK_X4), which was cultured separately at pH 4, and unacclimated *B. velezensis* (designated CK_WX7), cultured separately at pH 7 for 10 days were collected, serving as controls for *B. velezensis*. Furthermore, the interactions of *C. capsici* with both acclimated and unacclimated *B. velezensis* at pH 4 and pH 7 (designated X4_Z, X7_Z, WX4_Z, and WX7_Z) were collected. Control samples of *C. capsici* were also collected, including those cultured separately at pH 4 and pH 7 (designated CK4_Z and CK7_Z). Three samples were collected in each group. Under aseptic conditions, a sterile inoculating loop was used to transfer 0.2 g of *B. velezensis* colonies and *C. capsici* into a centrifuge tube. The samples were immediately frozen in liquid nitrogen and subsequently stored at −80℃ until RNA extraction.

Total RNA was extracted using Trizol reagent (Thermo Fisher, 15596018) in accordance with the manufacturer’s instructions. The quantity and purity of the total RNA were assessed using the Bioanalyzer 2100 and the RNA 6000 Nano LabChip Kit (Agilent, CA, USA, 5067-1511). High-quality RNA samples with an RIN (RNA Integrity Number) greater than 7.0 were utilized for the construction of the sequencing library. Following the extraction of total RNA, mRNA was purified from 5 µg of total RNA using Dynabeads Oligo (dT) (Thermo Fisher, CA, USA) through two rounds of purification. Subsequently, the purified mRNA was fragmented into short segments using divalent cations at elevated temperatures, specifically employing the Magnesium RNA Fragmentation Module (NEB, E6150, USA) at 94°C for 5–7 minutes. The cleaved RNA fragments were then reverse-transcribed to synthesize complementary DNA (cDNA) using SuperScript II Reverse Transcriptase (Invitrogen, 1896649, USA). This cDNA was subsequently used to synthesize U-labeled second-stranded DNAs with *E. coli* DNA polymerase I (NEB, catalog number M0209, USA), RNase H (NEB, M0297, USA), and dUTP Solution (Thermo Fisher, R0133, USA). The average insert size for the final cDNA libraries was 300 ± 50 bp. Finally, 2 × 150 bp paired-end sequencing (PE150) was performed on an Illumina NovaSeq 6000 (LC-Bio Technology Co., Ltd., Hangzhou, China) following the vendor’s recommended protocol.

### RNA-seq analysis

Cutadapt (version 1.9) was utilized to remove the reads that contained adaptor contamination, low-quality bases, and undetermined bases (14). Subsequently, the quality of the sequences was assessed using FastQC, which included evaluations of Q20, Q30, and the GC content of the cleaned data. All subsequent analyses were conducted using high-quality, clean data. *De novo* assembly of the transcriptome was executed with Trinity (version 2.15) (15). All assembled Unigenes were aligned against several databases, including the non-redundant (Nr) protein database, Gene Ontology (GO), SwissProt, the Kyoto Encyclopedia of Genes and Genomes (KEGG), and eggNOG databases using DIAMOND (version 2.0.15) with a threshold of Evalue <0.00001 (16–18). Salmon (version 1.9.0) was used to perform expression level for Unigenes by calculating TPM (Transcripts Per Kilobase of exon model per Million mapped reads). The differentially expressed Unigenes were selected with log_2_ greater than 1 or less than −1, with a false discovery rate (FDR) of less than 0.05, utilizing the R package edgeR (version 3.40.2) (19). To elucidate the functions of the differentially expressed genes, Gene Ontology functional enrichment and KEGG pathway analyses were conducted using Goatools and KOBAS, respectively (20). Protein-protein interactions were assessed.

### Quantitative real-time PCR

A total of 37 differentially expressed core genes identified by protein-protein interaction network analysis were selected for further quantitative real-time PCR (qRT-PCR) analysis. Each treatment group, which includes CK_WX7 vs CK_X4, WX4_X vs X4_X, and WX7_X vs X7_X, comprised 20 candidate genes. The housekeeping gene 16s RNA, which is widely recognized in the field, was chosen as the reference gene. The expression levels of the target mRNA were normalized against those of the reference gene, and the transcriptional levels of the various genes were analyzed and compared using the 2^−∆∆Ct^ method (21).

### Statistical analysis

All experiments were analyzed independently, and all statistical analyses were statistically analyzed using one-way analysis of variance (ANOVA) (*P* < 0.05). Protein–protein interactions were assessed utilizing the STRING database (Search Tool for the Retrieval of Interacting Genes/Proteins database, version 12.0) database (22). The resulting data were imported into Cytoscape (version 3.10.0), where the network analyzer function was employed to calculate the degree of interaction for each protein.

## RESULTS

### Acid resistance of *B. velezensis* and its antagonistic effects on *C. capsici*

The viable bacterial count of *B. velezensis* under acidic conditions, as shown in Table S2, demonstrated a gradual decrease with lowering pH. Notably, below pH 3, there are very few survival bacteria. We domesticated *B. velezensis* by gradually lowering the pH of the medium from 5.0 to 2.0. The viable bacterial counts of *B. velezensis* in the pH 5.0, 4.5, 4.0, and 3.5 treatments fluctuated but demonstrated a steady increase over five generations of successive passaging cultures. Notably, the viable count increased by 66.27% at pH 4.0. Based on these findings, we concluded that *B. velezensis* exhibits genetic stability under these pH conditions.

**Fig 1:**
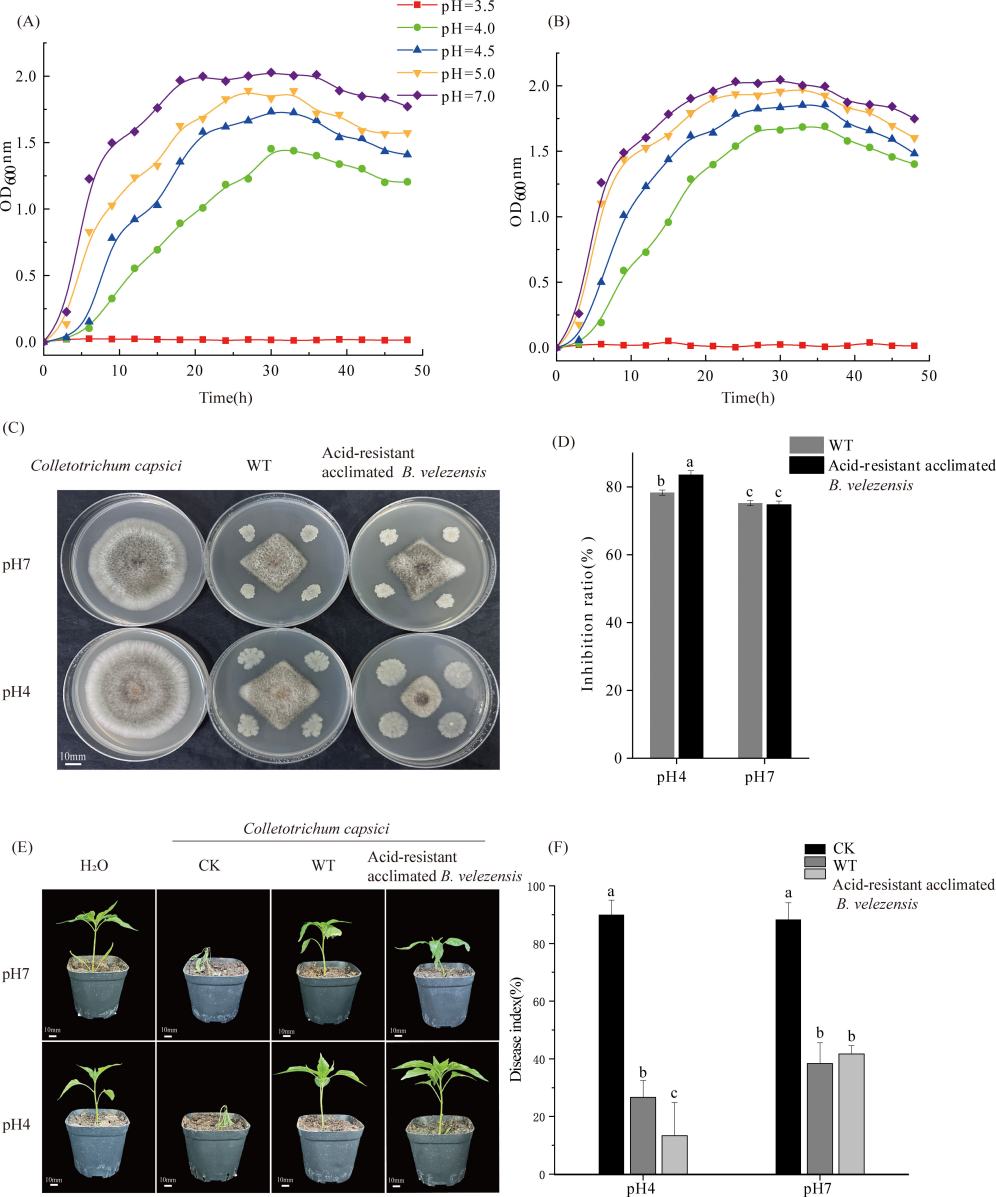
Growth characteristics of unacclimated (**A**) and acclimated *B. velezensis* (**B**) at different pH. (**C**) The mycelial morphology of *C. capsici* and *B. velezensis* (WT)/acid-resistant *B. velezensis* on PDA medium at pH 7 and pH 4. (**D**) Inhibition rate of *C. capsici* by *B. velezensis* after Day 7. Symptoms (**E**) and disease index (**F**) of pepper inoculated with *C. capsici* under different treatments. Values are shown as means ± standard error (SE).

We further analyzed the growth curves under these conditions. At pH 7.0 and 5.0, the original strain of *B. velezensis* exhibited a brief lag phase before rapidly progressing into the logarithmic growth phase, achieving peak OD_600_ of 2.02 and 1.89, respectively (Fig. 1A). By contrast, at pH 4.5 and 4.0, the growth rate of *B. velezensis* slowed, resulting in a lag phase of 6 hours, with maximum OD_600_ of 1.733 and 1.453, respectively. Notably, at pH 3.5, the lag phase extended to over 48 hours for *B. velezensis*. After acclimation (Fig. 1B), the growth trajectory of *B. velezensis* closely resembled that of the original strain, except at pH 3.5, where no significant variation in OD_600_ was observed. Intriguingly, at pH 4.5 and 4.0, *B. velezensis* entered the logarithmic growth phase an impressive 3 hours earlier than the original strain.

The mycelia of *C. capsici* treated with domesticated *B. velezensis* exhibited a smaller and more compact structure compared to untreated mycelia, and untreated mycelia had more extensive and circular growth patterns (Fig. 1C). On the 7th day of co-culture of *B. velezensis* with *C. capsici,* the highest inhibition observed was 83.55% by *B. velezensis* against *C. capsici* at pH 4, which was significantly greater than the inhibition at pH 7 (Fig. 1D). Furthermore, inoculating acid-resistant domesticated *B. velezensis* on Capsicum effectively defended against *Colletotrichum capsici* (Fig. 1E). The disease index of Capsicum inoculated with acid-resistant acclimated *B. velezensis* under pH 4 was markedly lower, showing reductions of 63.33% and 76.67% compared to the un-inoculated and WT treatments, respectively (Fig. 1F). At pH 7, the stem growth of acid-resistant acclimated *B. velezensis* appears to be visibly worse compared to other conditions. This may be attributed to the inability of the acid-resistant acclimated *B. velezensis* to quickly adapt to a neutral environment, resulting in a more favorable phenotype under acidic conditions.

### Differences in the gene expression of *B. velezensis* and *C. capsici*

To further elucidate the mechanisms underlying acid tolerance in the strain XY40-1, we employed RNA sequencing (RNA-Seq). Statistical analysis and quality assessment of the sequencing data revealed that *B. velezensis* and *C. capsici* produced a total of 194,245,280 and 785,557,232 raw reads, respectively (Tables S4 and S5). Following filtration, clean reads accounted for over 94% of the raw reads, with Q20 and Q30 values for each sample exceeding 90%. These results indicate that the sequencing quality of each sample was reliable. The results of the principal component analysis (PCA) diagram showed that the gene expression changes in *B. velezensis* and *C. capsici* were explained at 57.61% and 75.71%, respectively, with high reproducibility and small intergroup variability (Fig. S1).

**Fig 2:**
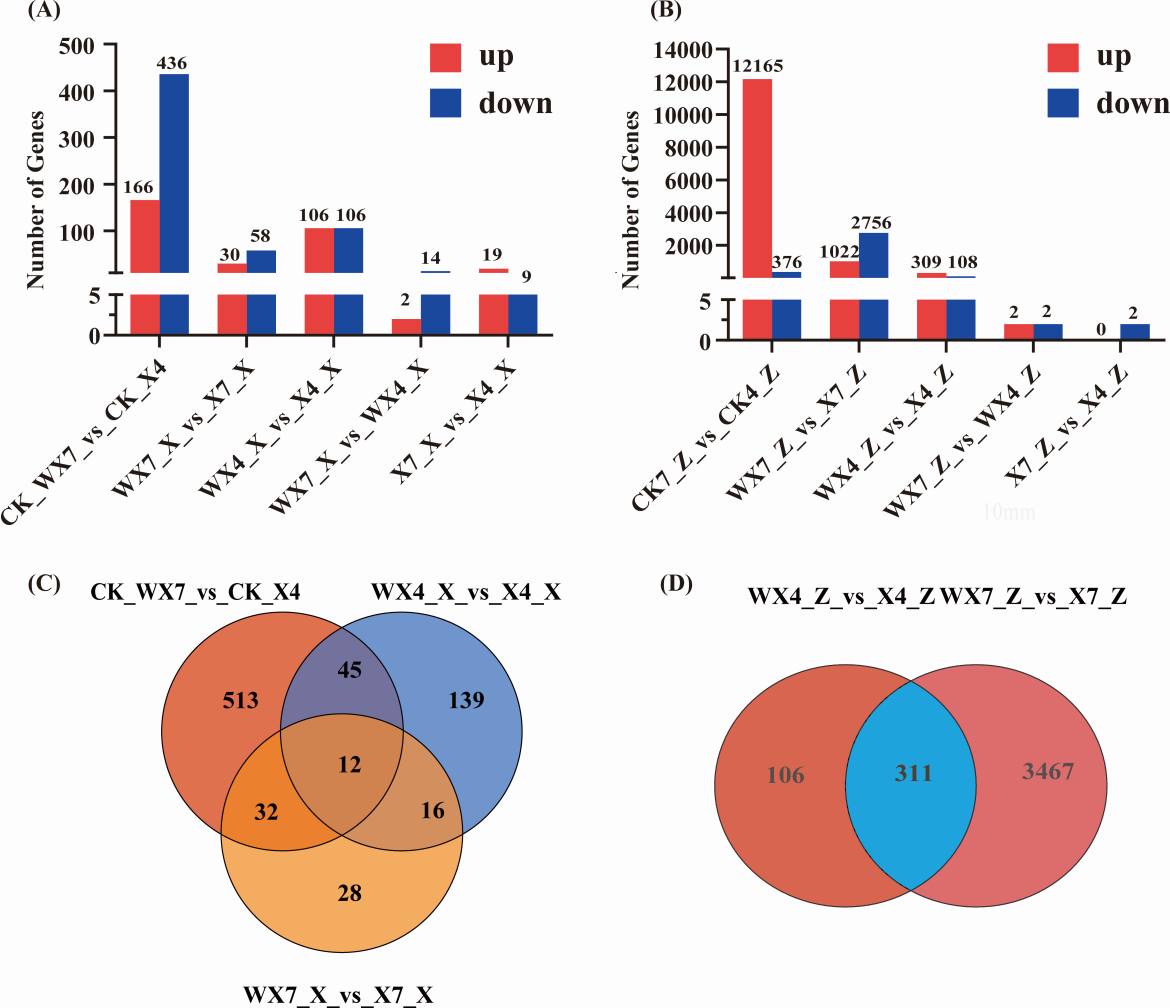
Total number of DEGs and the common and special DEGs in *B. velezensis* (**A, C**) and *C. capsici* (**B, D**).

In the comparison between the WX7_X vs WX4_X group and the X7_X vs X4_X group, the gene count of *B. velezensis* revealed 16 differentially expressed genes (DEGs) in the former group and 28 DEGs in the latter, as illustrated in Fig. 2A. These two groups exhibited a lower gene response compared to other groups, directing the focus towards the treatment groups before and after the domestication treatment. Notably, the abundance of DEGs at pH 4 was significantly greater than at pH 7, indicating a more pronounced antagonistic reaction of *B. velezensis* toward *C. capsici* under acidic conditions. *B. velezensis* activated numerous genes in response to *C. capsici* infection. Although there was overlap in differential gene expression between the control group and the treatment group (Fig. 2C), a detailed analysis unveiled distinct patterns of exclusive gene expression. Specifically, 513 genes were uniquely expressed in the CK_WX7 vs CK_ X4 comparison, 28 genes were exclusively present in the WX7_X vs X7_X group, and 139 genes were specific to the WX4_X vs X4_X group.

The gene count of *C. capsici* shows that a total of 12,541 differential genes were detected in the control group, with 12,165 genes being upregulated and 376 genes downregulated, as indicated in Fig. 2B. By contrast, the WX4_Z vs X4_Z group and WX7_Z vs X7_Z group had 417 and 4,778 differential genes, respectively. This disparity emphasizes significant differences in gene expression between the control and treatment groups, highlighting the impact of *B. velezensis* on *C. capsici*. Notably, Venn diagram analysis revealed that 106 and 3,467 genes were exclusively expressed in the WX4_Z vs X4_Z and WX7_Z vs X7_Z groups, respectively (Fig. 2D).

### Differences in the numbers of *B. velezensis* and *C. capsici*

**Fig 3:**
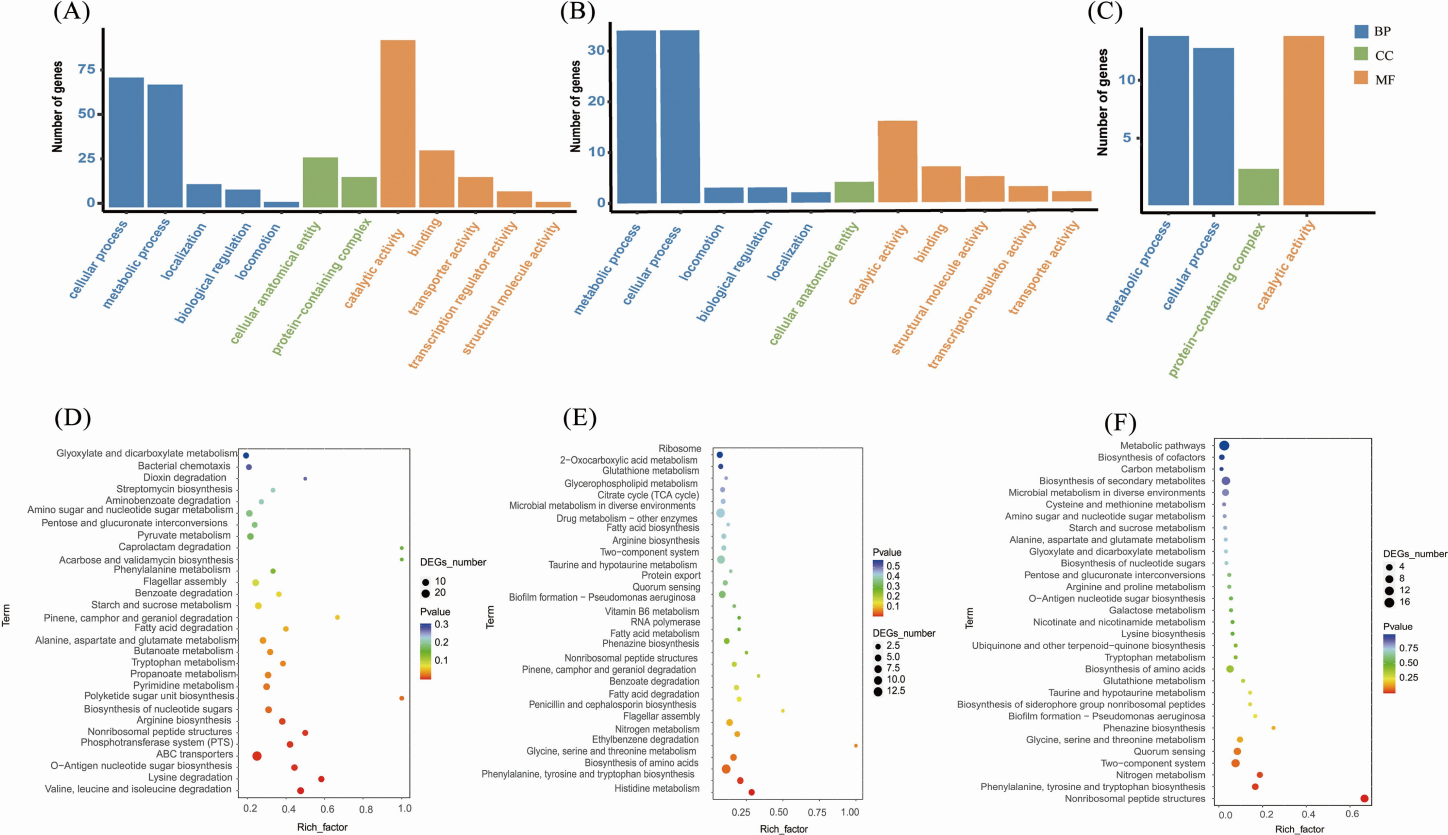
Global aspects of *B. velezensis* transcriptome. Histogram of GO functional enrichment analysis of DEGs in CK_WX7_vs_CK_X4 (**A**), WX4_X_vs_X4_X (B), and WX7_X_vs_X7_X (**C**) groups. Bubble chart of KEGG pathway enrichment analysis of DEGs in CK_WX7_vs_CK_X4 (**D**), WX4_X_vs_X4_X (**E**), WX7_X_vs_X7_X (**F**) groups.

**Fig 4:**
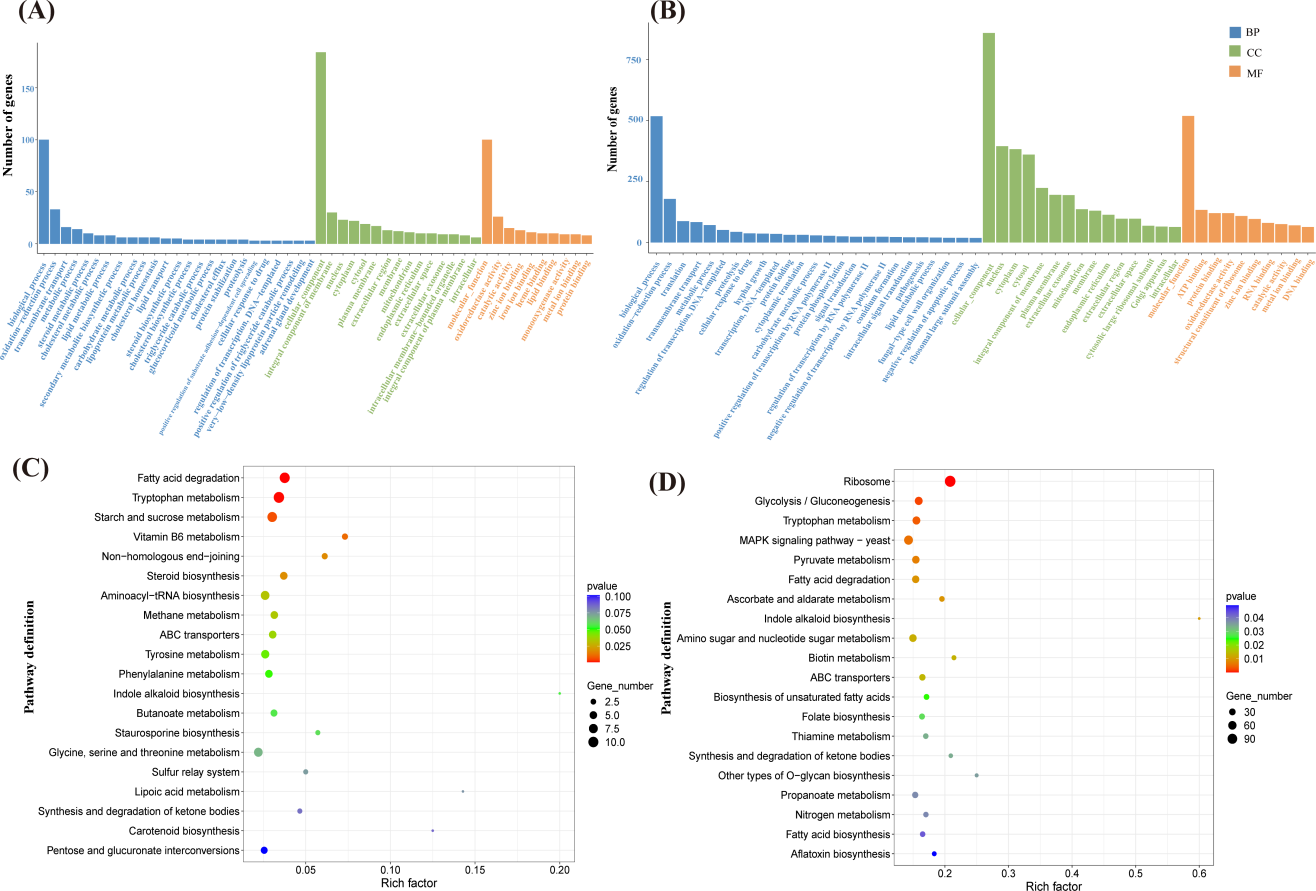
Global aspects of *C. capsici* transcriptome. Histogram of GO functional enrichment analysis of DEGs in WX4_Z_vs_X4_Z (**A**) and WX7_Z_vs_X7_Z (**B**) groups. Bubble chart of KEGG pathway enrichment analysis of DEGs in WX4_Z_vs_X4_Z (**C**) and WX7_Z_vs_X7_Z (**D**) groups.

The GO analysis categorized the unigenes into biological processes (BP), cellular components (CC), and molecular functions (MF). *B. velezensis* activates distinct genes in various treatments to counter *C. capsici* infection, with DEGs primarily enriched in BP and MF. Specifically, in the CK_WX7 vs CK_X4 group, DEGs were significantly enriched in catalytic activity, cellular processes, and metabolic processes. By contrast, DEGs involved in cellular and metabolic processes were enriched in WX4_X vs X4_X and WX7_X vs X7_X groups. These findings suggest that *B. velezensis* disrupts the normal growth of *C. capsici* mycelium and cells by modulating cellular and metabolic processes, thereby inhibiting fungal growth (Fig. 3A through C). For *C. capsici*, in BP, the WX4_Z_vs_X4_Z and WX7_Z_vs_X7_Z comparator groups exhibited the highest number of DEGs associated with the oxidation-reduction process. Following this, the WX4_Z_vs_X4_Z comparator group displayed a greater number of DEGs related to transmembrane transport, whereas the WX7_Z_vs_X7_Z comparator group showed a higher abundance of DEGs associated with translation. Concerning MF, the DEGs in the WX7_Z_vs_X7_Z group showed enrichment in ATP binding and protein binding, with the WX4_Z_vs_X4_Z comparative group further enriched in oxidoreductase activity and catalytic activity (Fig. 4A and B). As for CC, both sets of DEGs were predominantly enriched in the nucleus.

The KEGG enrichment analysis of *B. velezensis* was conducted on the differentially expressed genes. Among the hetero-expressed genes, 30 metabolic pathways with significant enrichment were selected for further investigation (Fig. 3D through F). The results revealed the ABC transporters pathway contained the highest number of differential genes, along with significant enrichment in valine, leucine, and isoleucine degradation pathways, lysine degradation, and O-antigen nucleotide sugar biosynthesis in the CK_WX7 vs CK_X4 group. In the WX4_X vs X4_X group, the differential genes were predominantly enriched in histidine metabolism, phenylalanine, tyrosine, and tryptophan biosynthesis, and the biosynthesis of amino acid pathways. In the WX7_X vs X7_X group, differential genes were mainly enriched in non-ribosomal peptide structures, phenylalanine, tyrosine, and tryptophan biosynthesis, as well as nitrogen metabolism pathways. For *C. capsici*, KEGG functional enrichment analysis identified the top 20 most significantly enriched pathways in each comparator group. Both comparator groups demonstrated enrichment in fatty acid degradation and tryptophan metabolism (Fig. 4C and D). In the WX4_Z_vs_X4_Z comparison group, differential genes were primarily enriched in starch and sucrose metabolism and vitamin B6 metabolism pathways. Similarly, in the WX7_Z_vs_X7_Z comparator group, differential genes were notably enriched in ribosome, glycolysis/gluconeogenesis, and pyruvate metabolism pathways.

### PPI network analysis

**Fig 5:**
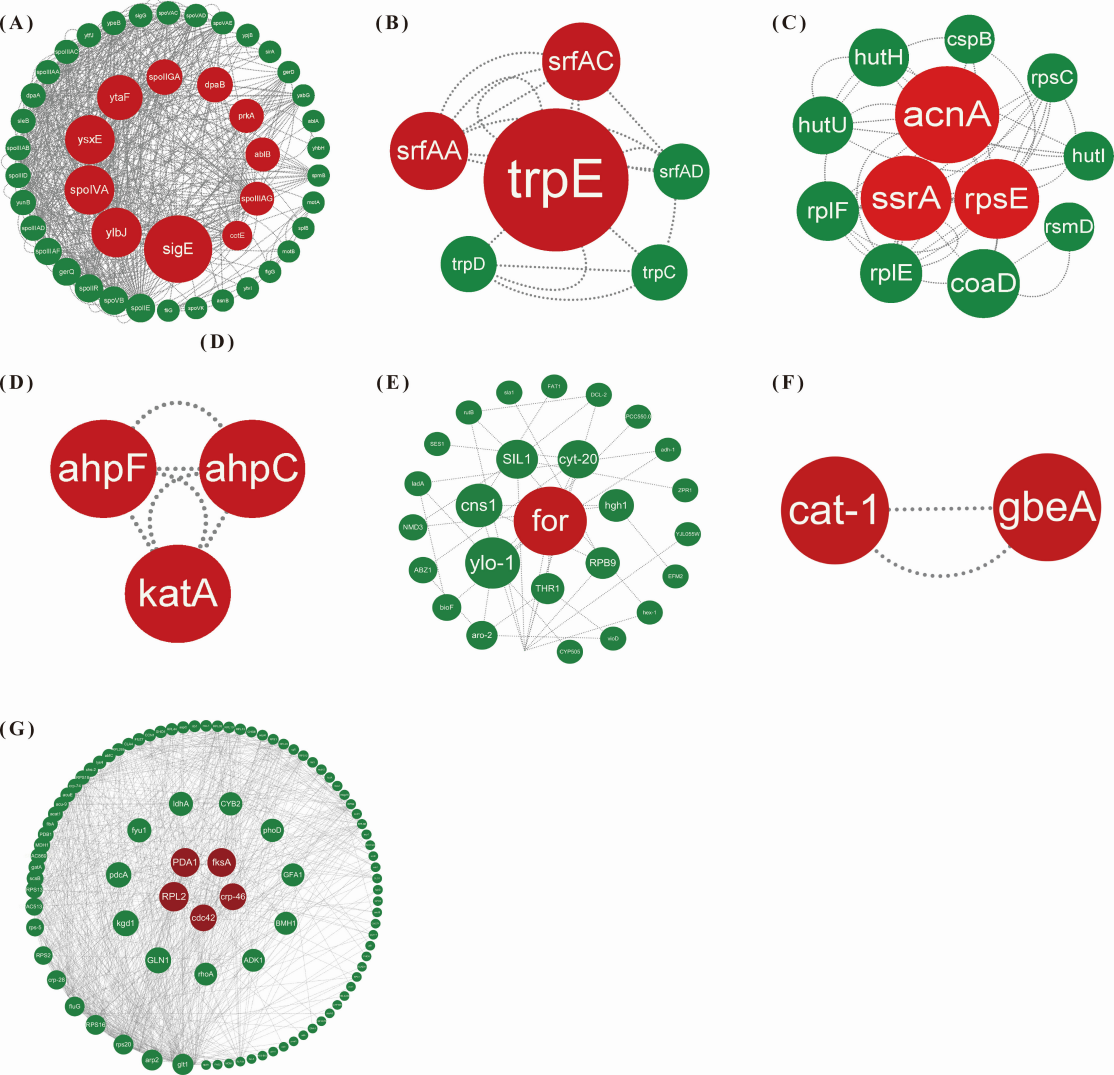
PPI network diagram of *B. velezensis* (**A–D**) and *C. capsica* (**E–G**). (**A**) Specific DEGs in CK_WX7_vs_CK_X4 group; (**B**) co-regulated DEGs in pH 7 and pH 4; (**C**) specific DEGs in WX4_X_vs_X4_X; (**D**) specific DEGs in WX7_X_vs_X7_X; (**E**) co-regulated DEGs in pH 7 and pH 4; (**F**) specific DEGs in WX4_Z_vs_X4_Z; and (**G**) specific DEGs in WX7_Z_vs_X7_Z.

**Fig 6:**
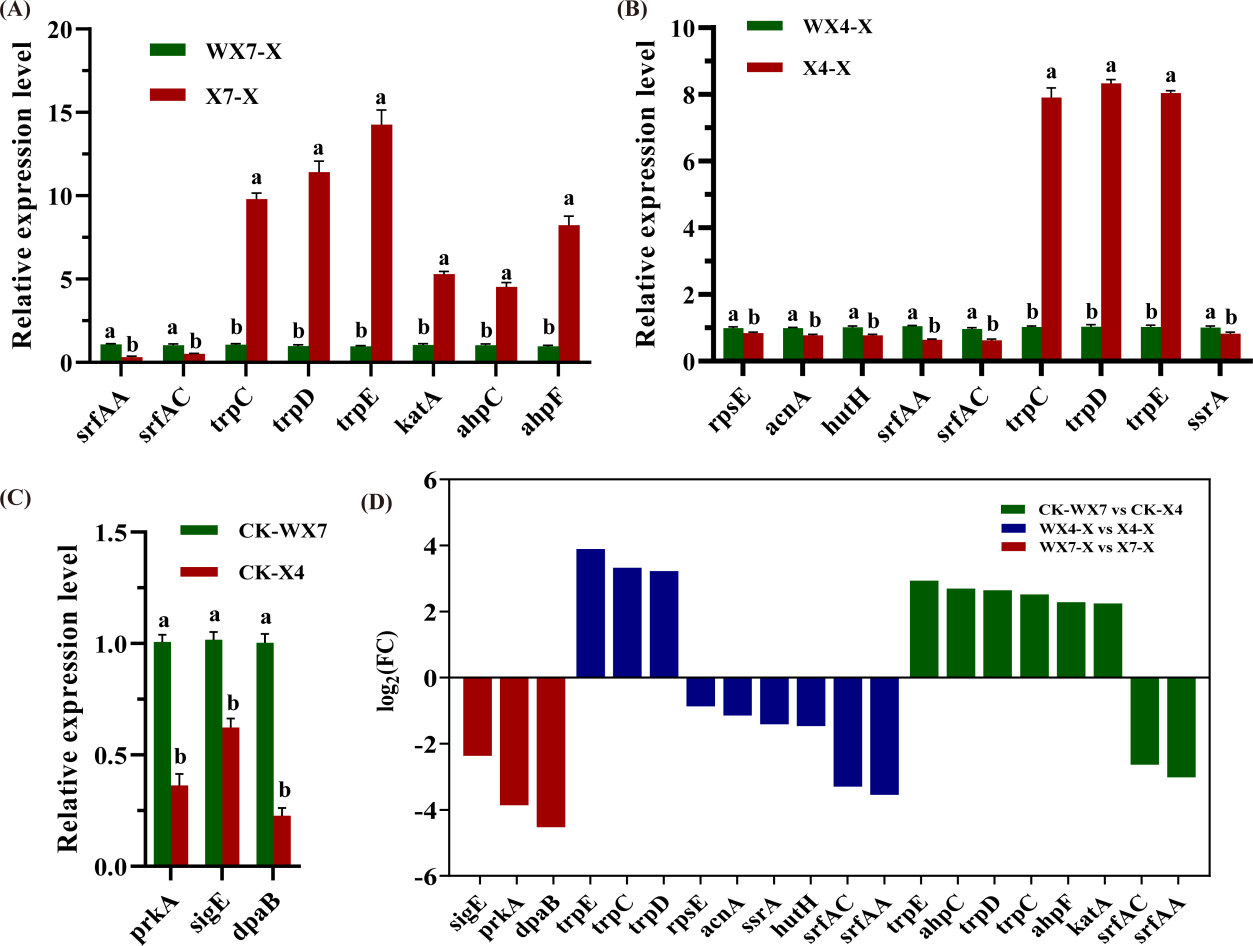
Validation of RNA-seq data by RT-qPCR analysis of *B. velezensis*. Relative expression levels of 15 core genes in the PPI network were determined by RT-qPCR (**A–C**) and compared with the results of RNA-seq (**D**).

We conducted a protein-protein interaction (PPI) network analysis on the genes exhibiting overlapping and specific differential expression as identified in the Venn diagram (Fig. 2). In the control group of *B. velezensis* (CK_WX7 vs CK_X4), specific core genes such as *sigE*, *dpaB*, and *PrkA,* which are primarily associated with endospore formation, were identified (Fig. 5A). Conversely, the core genes that they co-express, including *srfAA*, *srfAC*, *srfAD*, *trpC*, *trpD*, and *trpE,* were mainly enriched in pathways related to non-ribosomal peptide structures and the biosynthesis of phenylalanine, tyrosine, and tryptophan (Fig. 5B). In the WX4_X vs X4_X group, specific core genes such as *acnA*, *ssrA*, *rpsE*, and *HutH* were linked to the synthesis of amylocyclicin, histidine metabolism, and ribosome (Fig. 5C). In the WX7_X vs X7_X group, specific core genes such as *katA*, *ahpF*, and *ahpC*, which belong to the peroxiredoxin family, play a role in cell protection against oxidative stress by detoxifying peroxides (Fig. 5D). For *C. capsici*, the core genes that are co-expressed include *for ylo-1, SIL1*, and *cyt-20,* which were enriched in pathways such as glycine, serine and threonine metabolism, carotenoid biosynthesis, protein processing in endoplasmic reticulum, and ribosome. In the WX4_Z_vs_X4_Z group, specific core genes such as *cat-1 and gbeA* were enriched in tryptophan metabolism and starch and sucrose metabolism pathways (Fig. 5F). In the WX7_Z_vs_X7_Z group, specific core genes, including *RPL2, PDA1, fksA, crp-46, cdc42,* among others, were significantly enriched in pathways such as ribosome, glycolysis/gluconeogenesis, MAPK signaling pathway—yeast (Fig. 5G). The relative mRNA expression levels of 20 core genes in the PPI network were selected for qRT-PCR analysis to validate the reliability of DEGs data. As shown in Fig. 6, the expression levels of these genes analyzed by qRT-PCR were mainly in agreement with the data of RNA-seq. The qRT-PCR analysis results therefore confirmed that the data of RNA-seq were reliable.

## DISCUSSION

*B. velezensis* has emerged as a promising biocontrol microorganism due to its remarkable ability to synthesize secondary metabolites and its strong colonization capacity within plant tissues. This has garnered considerable attention in the field. However, soil acidification exacerbates the incidence of diseases in cash crops, including capsicum, ultimately impacting their economic viability (13). To address these challenges, we successfully domesticated and isolated a strain of *B. velezensis* capable of thriving at pH 4, with a growth trajectory closely mirroring that of the original strain (Fig. 1). Dormant spores in *B. velezensis* exhibit remarkable resistance when exposed to acidic environmental stresses, serving as a critical survival strategy that enables the bacteria to endure unfavorable conditions (23). In the study, the downregulation of spore formation-related genes, including *sigE, dpaB,* and *PrkA*, was observed under acidic conditions. Bacterial viable counts and spore counts are generally negatively correlated. We quantified viable bacteria counts of *B. velezensis* across five consecutive generations of passaged cultures subjected to pH 5.0, 4.5, 4.0, and 3.5 (Table S2). The results showed that there was a consistent increase in the number of viable bacteria at different pH, suggesting that the survival of *B. velezensis* under acidic conditions was enhanced by acid-tolerant domestication. Thus, we conjectured that the number of spores able to withstand unfavourable conditions was reduced, which was consistent with the results of the downregulation of genes related to spore formation.

The primary aim of this study was to elucidate the molecular mechanisms underlying the interaction between *B. velezensis* XY40-1 and *C. capsici*. Our findings demonstrated that *B. velezensis* substantially enhances its inhibitory effect on *C. capsici* and promotes crop health following acid-resistant domestication (Fig. 1). The antifungal strategy employed by *B. velezensis* is often multifaceted. Transcriptome analysis of *B. velezensis* in response to *C. capsici* revealed that the most significantly enriched metabolic pathways were related to nonribosomal peptide structures and the biosynthesis of phenylalanine, tyrosine, and tryptophan (Fig. 3). Surfactin, synthesized from nonribosomal peptide structures, exhibits stability across a broad pH range (24). Surfactin plays a key role in biocontrol by facilitating biofilm formation in *Bacillus*. By disrupting the biofilms of plant pathogens, this process can lead to cell damage and death, thereby inducing systemic resistance in plants (25). In the study, the expression of the *srfAA, srfAC,* and *srfAD* genes, which encode the *srfA* manipulator in the nonribosomal peptide pathway, was found to be downregulated (Fig. 5 and 6). Similarly, the downregulation of the *srfAD* gene in *B. velezensis* was observed following infection by *C. gloeosporioides* (20). Wang et al. showed that *B. velezensis* precisely regulates the expression of *srfAD* and related genes to synthesize surfactin and control the metabolic pathway in response to *C. gloeosporioides* infections (20). This suggests that despite a reduction in surfactin production, it still exhibited a significant antagonistic effect on *C. capsici*. Moreover, the core genes *trpE* and *trpD* were upregulated in *B. velezensis* (Fig. 5 and 6), playing a critical role in the regulation of phenylalanine, tyrosine, and tryptophan biosynthesis. Among these, tryptophan serves as a precursor for the production of IAA through enzymatic conversion. The upregulation of the *trpC* gene further supports this process, as it directly participates in IAA biosynthesis. Zaid DS et al. (26) identified the genes *trpA, trpB, trpC, trpD, trpE,* and *trpF* in the *Bacillus velezensis* HNA3 genome as being involved in IAA production. The increase in IAA promotes the growth and root development of *Capsicum*. These findings suggest that *B. velezensis* was involved in IAA synthesis through amino acid metabolism in response to *C. capsici*.

In co-cultured PDA agar plates, *C. capsici* growth was inhibited and exhibited rhombus-shaped morphology (Fig. 1). Transcriptomic analysis revealed that PPI core genes were associated with glycine, serine and threonine metabolism, carotenoid biosynthesis, ribosome, and protein processing in endoplasmic reticulum pathways (Fig. 4). For instance, the downregulation of the gene (*for*) encoding serine hydroxymethyltransferase (SHMT) can have significant implications for both *C. capsici* and *B. velezensis*. First, reduced SHMT activity may result in decreased accumulation of serine, glycine, and 5,10-methylene THF (27). For *C. capsici*, this condition affects nucleotide synthesis, leading to a slowing down of fungal growth in response to bacterial attack. Meanwhile, for *B. velezensis,* serine is an essential amino acid for bacterial growth, and the reduction of glycine may limit the utilization of nitrogen sources by *B. velezensis*, thereby inhibiting its proliferation. Furthermore, previous studies have shown that serine has antioxidant and cytoprotective effects on HUVECs by inducing antioxidant factors such as Nrf2, HO-1, and NO (28). Although the relationship between SHMT and ROS resistance in *C. capsici* has not been explicitly studied, it is plausible to hypothesize that serine could enhance fungal resistance to reactive oxygen species (ROS) generated by *B. velezensis*. In addition, carotenoids produced through the upregulation of *ylo-1* possess antioxidant properties and can enhance self-resistance by scavenging DPPH radicals, reducing antioxidant capacity, and inhibiting lipid peroxidation activity (29). Furthermore, the downregulation of *cns1*, a gene involved in the ribosomal pathway, and *sil1*, a gene associated with protein processing in the endoplasmic reticulum, suggests a decline in mitochondrial function and endoplasmic reticulum homeostasis in *C. capsici* cells (30, 31), which weakens its defense mechanisms (Fig. 5 and 6). Overall, *C. capsici* likely defends itself against *B. velezensis* through oxidative stress response, restriction of bacterial nutrient competition, and maintenance of cellular homeostasis.

Under acidic conditions, despite the downregulation of certain core genes in *B. velezensis*, this bacterium continued to exhibit significant antagonistic activity against *C. capsici*. Notably, genes such as a*cnA, ssrA, rpsE,* and *HutH* are involved in the synthesis of amylocyclicin, histidine metabolism, and ribosomal function (Fig. 5 and 6). *B. velezensis* is known for producing various antibacterial compounds, including ribosomally synthesized amylocyclicin, as demonstrated by multiple studies (32, 33). In this study, the downregulation of the *ssrA* and *rpsE* genes, crucial for ribosome function, may have impacted amyocyclin synthesis (34). Consequently, *acnA*, a gene involved in amyocyclin synthesis, was also downregulated. By contrast, previous research has reported an upregulation of *acnA* in *B. velezensis* during antifungal activity (35). Despite these discrepancies in gene expression patterns, both findings emphasize the capacity of *B. velezensis* to finely tune the expression levels of *acnA* and related genes, thereby regulating amylocyclicin synthesis and modulating this metabolic pathway in response to pathogen-induced stress. Furthermore, the *HutH* gene plays a crucial role in histidine metabolism, which is essential for bacterial adaptation in acidic environments (36, 37). These findings collectively suggest that amylocyclicin production, histidine metabolism, and ribosomal function are significant factors in the response of *B. velezensis* to *C. capsici*. On the other hand, *C. capsici* resists *B. velezensis* primarily through pathways related to tryptophan metabolism and starch and sucrose metabolism (Fig. 4). Transcriptome analysis revealed a significant upregulation of the *cat-1* gene, which is involved in the tryptophan metabolism pathway and encodes peroxisomes in *C. capsici* treated with B. velezensis. Previous studies have demonstrated that genes encoding peroxidases in C. orbiculare (responsible for melon plant anthracnose) are integral to the formation and proliferation of peroxisomes (38), which are closely linked to chitin synthesis and the maintenance of mycelial cell wall integrity (39). Zhang J et al. (40) discovered that *Fusarium graminearum* combats *B. velezensis* YB185 infection by activating peroxisomes. It is hypothesized that by enhancing peroxisome formation, the mycelium can repair the mycelial cell membrane, synthesize chitin, and ensure cell wall integrity. In addition, the *gbeA* gene, which encodes glucan, is crucial for the synthesis of starch and glycogen and contributes to cell wall synthesis, thereby increasing resistance to bacterial attack (41) (Fig. 5 and 6). In summary, under acidic conditions, *C. capsici* engages multiple metabolic pathways to preserve cell wall integrity, enhances carbohydrate utilization efficiency, and boosts its energy production. These adaptations enhance its defensive capabilities, enabling it to effectively compete with *B. velezensis*.

Under neutral conditions, the defensive response of *B. velezensis* to *C. capsici* may involve the upregulation of the *katA*, *ahpF*, and *ahpC* genes, which belong to the peroxiredoxin family (Fig. 5 and 6). This upregulation may serve as a strategy employed by *B. velezensis* to mitigate the oxidative stress response induced by *C. capsici* or triggered by their interaction. By enhancing the expression of these antioxidant enzymes, *B. velezensis* can more effectively eliminate reactive oxygen species (ROS) generated during their interaction (42). This mechanism helps protect the cellular components of *B. velezensis*, reducing damage caused by ROS and thereby providing an advantage in symbiotic or competitive relationships among microorganisms. *C. capsici* exhibited the highest number of differentially expressed genes under neutral conditions, indicating a strategic adjustment of its metabolic activities to enhance its adaptive capacity (Fig. 2). Analysis of metabolic pathways, based on KEGG functional annotation and PPI core gene enrichment, identified the ribosome pathway as a key component. Specific pathways involved include glycolysis/gluconeogenesis and MAPK signaling pathway—yeast pathway (Fig. 4). Specifically, the gene *PDA1* involved in glycolysis was found to be downregulated. Gao et al. (43) identified a secreted polysaccharide deacetylase (*PDA1*) from the soil-borne fungus *Verticillium dahliae,* which promotes virulence by directly deacetylating chitin oligomers—a component widely present in the fungus. Consequently, it is hypothesized that under co-culture conditions, the virulence of *C. capsici* is attenuated. In addition, the gene *cdc42* activates the MAPK signaling pathway—yeast pathway, regulates cell polarized growth, and enables *C. capsici* to adapt to *B. velezensis* infiltration (44) (Fig. 5 and 6).

**Fig 7:**
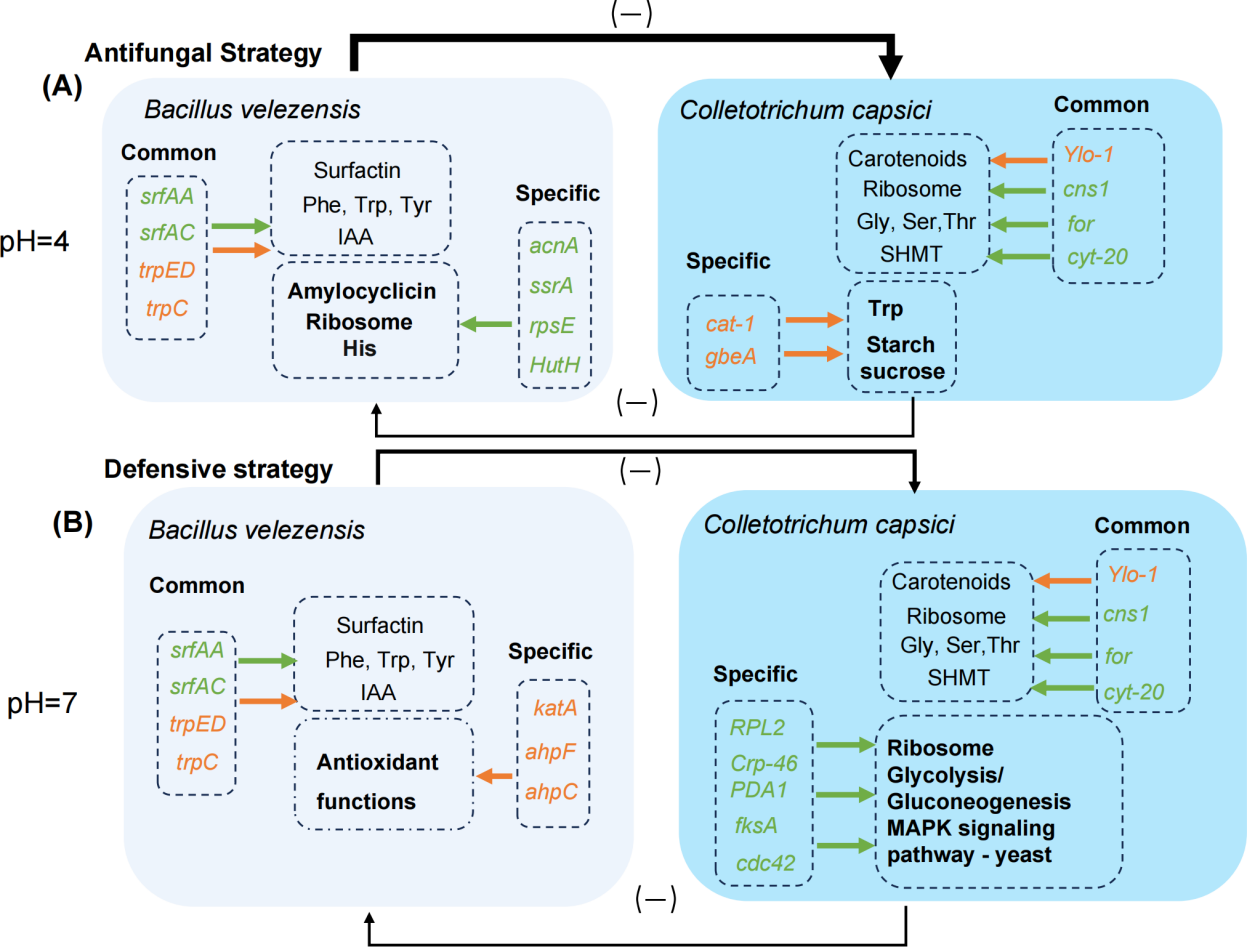
Schematic diagram of common and specific genes involved in acid-resistant *B. velezensis* and *C. capsici* interaction under pH 4 (**A**) and pH 7 (**B**) dual cultures. Orange indicates upregulated genes, while green signifies downregulated genes.

In total, the interaction between *B. velezensis* and *C. capsici* significantly influences the survival and reproduction of both organisms through various mechanisms. These mechanisms include direct antibacterial effects, promotion of plant growth, enhancement of amino acid and lipid metabolism, and improvement of defense capabilities, all of which contribute to biological control effects. Specifically, domesticated *B. velezensis* employs a direct antifungal strategy under acidic conditions to strengthen its inhibitory effect, while in neutral conditions, it prioritizes the enhancement of its defense mechanisms (Fig. 7). Consequently, domesticated *B. velezensis* demonstrates improved biological control effects in acidic environments. These discoveries not only substantiate the utilization of *B. velezensis* as a biological control agent but also highlight its prospective applications within the agricultural and environmental sectors.

### Conclusion

*B. velezensis* employs various synergistic mechanisms to effectively control *C. capsici*. It primarily defends against *C. capsici* through the regulation of the synthesis of surfactin, the metabolism of amino acids (e.g., phenylalanine, tyrosine, and tryptophan), and IAA. *C. capsici* utilizes several strategies, including carotenoid synthesis, ribosome, protein processing in the endoplasmic reticulum, and amino acid metabolic pathways, to defend against or inhibit the growth of *B. velezensis*. The acid-tolerant strain *B. velezensis* XY40-1 exhibits significant antagonistic activity against *C. capsici* in acidic dual cultures. This antagonism is facilitated by the synthesis of amylocyclicin and the metabolism of ribosomes and histidine. In response, *C. capsici* maintains cell wall integrity by utilizing tryptophan metabolism and starch and sucrose metabolic pathways to enhance its resistance. In neutral conditions, *B. velezensis* activates its defense mechanisms against *C. capsici* through the production of catalase. Concurrently, *C. capsici* adapts to the infiltration by *B. velezensis* by regulating glycolysis and the MAPK signaling pathway—yeast pathway, which supports and maintains its polarized cell growth.

## Data Availability

The data presented in the study were deposited in the National Center for Biotechnology Information (NCBI) database under BioProject ID PRJNA1261628.

## SUPPLEMENTAL MATERIAL

Supplemental material aem.00340-25-s0001.docx Tables S1 to S4; Fig. S1.

ASM does not own the copyrights to Supplemental Material that may be linked to, or accessed through, an article. The authors have granted ASM a non-exclusive, world-wide license to publish the Supplemental Material files. Please contact the corresponding author directly for reuse.

## References

[1] Liu F, Zhao J, Sun H, Xiong C, Sun X, Wang X, Wang Z, Jarret R, Wang J, Tang B, et al.. 2023. Genomes of cultivated and wild Capsicum species provide insights into pepper domestication and population differentiation. Nat Commun 14:5487. doi:10.1038/s41467-023-41251-4 37679363 PMC10484947

[2] Ruangwong O-U, Pornsuriya C, Pitija K, Sunpapao A. 2021. Biocontrol mechanisms of Trichoderma koningiopsis PSU3-2 against postharvest anthracnose of chili pepper. J Fungi 7:276. doi:10.3390/jof7040276 PMC8067587 33916921

[3] Moreno-Gavíra A, Diánez F, Sánchez-Montesinos B, Santos M. 2021. Biocontrol effects of Paecilomyces variotii against fungal plant diseases. J Fungi (Basel) 7:415. doi:10.3390/jof7060415 34073454 PMC8228970

[4] Chen XH, Koumoutsi A, Scholz R, Eisenreich A, Schneider K, Heinemeyer I, Morgenstern B, Voss B, Hess WR, Reva O, Junge H, Voigt B, Jungblut PR, Vater J, Süssmuth R, Liesegang H, Strittmatter A, Gottschalk G, Borriss R. 2007. Comparative analysis of the complete genome sequence of the plant growth-promoting bacterium Bacillus amyloliquefaciens FZB42. Nat Biotechnol 25:1007–1014. doi:10.1038/nbt1325 17704766

[5] Chowdhury SP, Hartmann A, Gao X, Borriss R. 2015. Biocontrol mechanism by root-associated Bacillus amyloliquefaciens FZB42 - a review. Front Microbiol 6:780. doi:10.3389/fmicb.2015.00780 26284057 PMC4517070

[6] Rabbee MF, Ali MS, Choi J, Hwang BS, Jeong SC, Baek K-H. 2019. Bacillus velezensis: a valuable member of bioactive molecules within plant microbiomes. Molecules 24:1046. doi:10.3390/molecules24061046 30884857 PMC6470737

[7] Kim YS, Lee Y, Cheon W, Park J, Kwon H-T, Balaraju K, Kim J, Yoon YJ, Jeon Y. 2021. Characterization of Bacillus velezensis AK-0 as a biocontrol agent against apple bitter rot caused by Colletotrichum gloeosporioides. Sci Rep 11:626. doi:10.1038/s41598-020-80231-2 33436839 PMC7804190

[8] Chen Z, Wang Z, Xu W. 2024. Bacillus velezensis WB induces systemic resistance in watermelon against Fusarium wilt. Pest Manag Sci 80:1423–1434. doi:10.1002/ps.7873 37939121

[9] Morán-Diez E, Rubio B, Domínguez S, Hermosa R, Monte E, Nicolás C. 2012. Transcriptomic response of Arabidopsis thaliana after 24 h incubation with the biocontrol fungus Trichoderma harzianum. J Plant Physiol 169:614–620. doi:10.1016/j.jplph.2011.12.016 22317785

[10] Wang K, Wang Z, Xu W. 2022. Induced oxidative equilibrium damage and reduced toxin synthesis in Fusarium oxysporum f. sp. niveum by secondary metabolites from Bacillus velezensis WB. FEMS Microbiol Ecol 98:fiac080. doi:10.1093/femsec/fiac080 35776952

[11] Liang N, Charron J-B, Jabaji S. 2023. Comparative transcriptome analysis reveals the biocontrol mechanism of Bacillus velezensis E68 against Fusarium graminearum DAOMC 180378, the causal agent of Fusarium head blight. PLoS One 18:e0277983. doi:10.1371/journal.pone.0277983 36701319 PMC9879434

[12] Huang S, Zhang W, Yu X, Huang Q. 2010. Effects of long-term fertilization on corn productivity and its sustainability in an Ultisol of southern China. Agric Ecosyst Environ 138:44–50. doi:10.1016/j.agee.2010.03.015

[13] Li X, Chen D, Carrión VJ, Revillini D, Yin S, Dong Y, Zhang T, Wang X, Delgado-Baquerizo M. 2023.Acidification suppresses the natural capacity of soil microbiome to fight pathogenic Fusarium infections. Nat Commun 14:6188. doi:10.1038/s41467-023-41564-4 37607924 PMC10444831

[14] Martin M. 2011. CUTADAPT removes adapter sequences from high-throughput sequencing reads. EMBnet j 17:10. doi:10.14806/ej.17.1.200

[15] Grabherr MG, Haas BJ, Yassour M, Levin JZ, Thompson DA, Amit I, Adiconis X, Fan L, Raychowdhury R, Zeng Q, Chen Z, Mauceli E, Hacohen N, Gnirke A, Rhind N, di Palma F, Birren BW, Nusbaum C, Lindblad-Toh K, Friedman N, Regev A. 2011. Full-length transcriptome assembly from RNA-Seq data without a reference genome. Nat Biotechnol 29:644–652. doi:10.1038/nbt.1883 21572440 PMC3571712

[16] Buchfink B, Xie C, Huson DH. 2015. Fast and sensitive protein alignment using DIAMOND. Nat Methods 12:59–60. doi:10.1038/nmeth.3176 25402007

[17] Patro R, Duggal G, Love MI, Irizarry RA, Kingsford C. 2017. Salmon provides fast and bias-aware quantification of transcript expression. Nat Methods 14:417–419. doi:10.1038/nmeth.4197 28263959 PMC5600148

[18] Mortazavi A, Williams BA, McCue K, Schaeffer L, Wold B. 2008. Mapping and quantifying mammalian transcriptomes by RNA-Seq. Nat Methods 5:621–628. doi:10.1038/nmeth.1226 18516045 PMC13303166

[19] Robinson MD, McCarthy DJ, Smyth GK. 2010. edgeR: a bioconductor package for differential expression analysis of digital gene expression data. Bioinformatics 26:139–140. doi:10.1093/bioinformatics/btp616 19910308 PMC2796818

[20] Wang L, Zhu T. 2023. Strong opponent of walnut anthracnose-Bacillus velezensis and its transcriptome analysis. Microorganisms 11:1885. doi:10.3390/microorganisms11081885 37630445 PMC10456653

[21] Livak KJ, Schmittgen TD. 2001. Analysis of relative gene expression data using real-time quantitative PCR and the 2^−ΔΔ*C*_T_^ method. Methods 25:402–408. doi:10.1006/meth.2001.1262 11846609

[22] Szklarczyk D, Gable AL, Nastou KC, Lyon D, Kirsch R, Pyysalo S, Doncheva NT, Legeay M, Fang T, Bork P, Jensen LJ, von Mering C. 2021. Correction to “The STRING database in 2021: customizable protein-protein networks, and functional characterization of user-uploaded gene/measurement sets”. Nucleic Acids Res 49:10800. doi:10.1093/nar/gkab835 34530444 PMC8501959

[23] Nicholson WL, Munakata N, Horneck G, Melosh HJ, Setlow P. 2000. Resistance of Bacillus endospores to extreme terrestrial and extraterrestrial environments. Microbiol Mol Biol Rev 64:548–572. doi:10.1128/MMBR.64.3.548-572.2000 10974126 PMC99004

[24] Li MSM, Piccoli DA, McDowell T, MacDonald J, Renaud J, Yuan Z-C. 2021. Evaluating the biocontrol potential of Canadian strain Bacillus velezensis 1B-23 via its surfactin production at various pHs and temperatures. BMC Biotechnol 21:31. doi:10.1186/s12896-021-00690-x 33926450 PMC8082884

[25] Wang L, Sha Y, Wu D, Wei Q, Chen D, Yang S, Jia F, Yuan Q, Han X, Wang J. 2020. Surfactant induces ROS-mediated cell membrane permeabilization for the enhancement of mannatide production. Process Biochem 91:172–180. doi:10.1016/j.procbio.2019.12.009

[26] Zaid DS, Cai S, Hu C, Li Z, Li Y. 2022. Comparative genome analysis reveals phylogenetic identity of Bacillus velezensis HNA3 and genomic insights into its plant growth promotion and biocontrol effects. Microbiol Spectr 10:e02169–21. doi:10.1128/spectrum.02169-21 35107331 PMC8809340

[27] Santatiwongchai J, Gleeson D, Gleeson MP. 2019. Theoretical evaluation of the reaction mechanism of serine hydroxymethyl transferase. J Phys Chem B 123:407–418. doi:10.1021/acs.jpcb.8b10196 30522268

[28] Maralani MN, Movahedian A, Javanmard SH. 2012. Antioxidant and cytoprotective effects of L-Serine on human endothelial cells. Res Pharm Sci 7:209–215. 23248671 PMC3523412

[29] Alonso-Villegas R, González-Amaro RM, Figueroa-Hernández CY, Rodríguez-Buenfil IM. 2023. The genus Capsicum: a review of bioactive properties of its polyphenolic and capsaicinoid composition. Molecules 28:4239. doi:10.3390/molecules28104239 37241977 PMC10224380

[30] Ma S, Yang W, Liu X, Li S, Li Y, Zhu J, Zhang C, Lu X, Zhou X, Chen R. 2022. Pentatricopeptide repeat protein CNS1 regulates maize mitochondrial complex III assembly and seed development. Plant Physiol 189:611–627. doi:10.1093/plphys/kiac086 35218364 PMC9157079

[31] Ichhaporia VP, Hendershot LM. 2021. Role of the HSP70 co-chaperone SIL1 in health and disease. Int J Mol Sci 22:1564. doi:10.3390/ijms22041564 33557244 PMC7913895

[32] Berezhnaya AV, Evdokimova OV, Valentovich LN, Sverchkova NV, Titok MA, Kolomiyets EI. 2019. Molecular genetic and functional analysis of the genome of bacteria Bacillus velezensis BIM B-439D. Appl Biochem Microbiol 55:386–396. doi:10.1134/S0003683819040033

[33] Gao X-Y, Liu Y, Miao L-L, Li E-W, Sun G-X, Liu Y, Liu Z-P. 2017. Characterization and mechanism of anti-Aeromonas salmonicida activity of a marine probiotic strain, Bacillus velezensis V4. Appl Microbiol Biotechnol 101:3759–3768. doi:10.1007/s00253-017-8095-x 28074223

[34] Keiler KC. 2015. Mechanisms of ribosome rescue in bacteria. Nat Rev Microbiol 13:285–297. doi:10.1038/nrmicro3438 25874843

[35] Rungsirivanich P, Parlindungan E, O’Connor PM, Field D, Mahony J, Thongwai N, van Sinderen D. 2021. Simultaneous production of multiple antimicrobial compounds by Bacillus velezensis ML122-2 isolated from Assam tea leaf [Camellia sinensis var. assamica (J.W.Mast.) Kitam.]. Front Microbiol 12:789362. doi:10.3389/fmicb.2021.789362 34899671 PMC8653701

[36] Bender RA. 2012. Regulation of the histidine utilization (hut) system in bacteria. Microbiol Mol Biol Rev 76:565–584. doi:10.1128/MMBR.00014-12 22933560 PMC3429618

[37] Zhang X-X, Rainey PB. 2007. Genetic analysis of the histidine utilization (hut) genes in Pseudomonas fluorescens SBW25. Genetics 176:2165–2176. doi:10.1534/genetics.107.075713 17717196 PMC1950622

[38] Kubo Y, Fujihara N, Harata K, Neumann U, Robin GP, O’Connell R. 2015. Colletotrichum orbiculare FAM1 encodes a novel woronin body-associated pex22 peroxin required for appressorium-mediated plant infection. mBio 6:e01305-15. doi:10.1128/mBio.01305-15 26374121 PMC4600112

[39] Bhambra GK, Wang Z-Y, Soanes DM, Wakley GE, Talbot NJ. 2006. Peroxisomal carnitine acetyl transferase is required for elaboration of penetration hyphae during plant infection by Magnaporthe grisea. Mol Microbiol 61:46–60. doi:10.1111/j.1365-2958.2006.05209.x 16824094

[40] Zhang J, Zhu W, Goodwin PH, Lin Q, Xia M, Xu W, Sun R, Liang J, Wu C, Li H, Wang Q, Yang L. 2022. Response of Fusarium pseudograminearum to biocontrol agent Bacillus velezensis YB-185 by phenotypic and transcriptome analysis. J Fungi (Basel) 8:763. doi:10.3390/jof8080763 35893131 PMC9331925

[41] Xin C, Ban X, Gu Z, Li C, Cheng L, Hong Y, Li Z. 2019. Non-classical secretion of 1,4-alpha-glucan branching enzymes without signal peptides in Escherichia coli. Int J Biol Macromol 132:759–765. doi:10.1016/j.ijbiomac.2019.04.002 30953720

[42] Qian J-M, Bai Y. 2021. Stuck on you: bacterial-auxin-mediated bacterial colonization of plant roots. Cell Host Microbe 29:1471–1473. doi:10.1016/j.chom.2021.09.014 34648737

[43] Gao F, Zhang B-S, Zhao J-H, Huang J-F, Jia P-S, Wang S, Zhang J, Zhou J-M, Guo H-S. 2019. Deacetylation of chitin oligomers increases virulence in soil-borne fungal pathogens. Nat Plants 5:1167–1176. doi:10.1038/s41477-019-0527-4 31636399

[44] Pérez P, Soto T, Gómez-Gil E, Cansado J. 2020. Functional interaction between Cdc42 and the stress MAPK signaling pathway during the regulation of fission yeast polarized growth. Int Microbiol 23:31–41. doi:10.1007/s10123-019-00072-6 30989357

