# Supplementary material for: Acid-resistant Bacillus velezensis effectively controls pathogenic Colletotrichum capsici and improves plant health through metabolic interactions
Source: Appl Environ Microbiol. 2025 Jun 23;91(7):e00340-25. doi: 10.1128/aem.00340-25 (PMC12285265; doi:10.1128/aem.00340-25)
Supplement: Supplemental material — Tables S1 to S4; Fig. S1. [file aem.00340-25-s0001.docx]

Supplementary material

Table S1 The soil pH before and after cultivation

| Treatment | Initial pH | After 30d pH | After 90d pH |
| --- | --- | --- | --- |
| pH4 | 6.00 | 4.37±0.12 | 4.13±0.06 |
| pH7 |  | 6.93±0.12 | 7.17±0.15 |

Table S2 The viable bacterial counts of *B. velezensis* during domestication at different pH

| pH |  | Viable count (10^9^CFU/mL) | | | | |  |
| --- | --- | --- | --- | --- | --- | --- | --- |
|  | Acid resistant before acclimation | The first generation | The second generation | The third generation | The fourth generation | The fifth generation | Percentage/% |
| 7.0（CK) |  | 1.96±0.04 | | | | |  |
| 5.0 | 1.55±0.01 | 1.59±0.01 | 1.6±0.02 | 1.63±0.02 | 1.68±0.01 | 1.7±0.02 | 9.68% |
| 4.5 | 1.25±0.01 | 1.30±0.04 | 1.34±0.02 | 1.41±0.06 | 1.53±0.04 | 1.58±0.03 | 26.40% |
| 4.0 | 0.83±0.04 | 0.91±0.07 | 1.03±0.10 | 1.28±0.01 | 1.33±0.04 | 1.38±0.02 | 66.27% |
| 3.5 | 0.55±0.04 | 0.61±0.03 | 0.65±0.01 | 0.67±0.01 | 0.71±0.03 | 0.83±0.07 | 50.90% |
| 3.0 | 0 | 0 | 0 | 0 | 0 | 0.05±0.03 | - |
| 2.5 | 0 | 0 | 0 | 0 | 0 | 0.015±0.02 | - |
| 2.0 | 0 | 0 | 0 | 0 | 0 | 0 | - |

Note: Due to instrument sensitivity limitations, bacterial counts below the detection threshold are recorded as 0.

Table S3 Sequencing Data Statistics of *B. velezensis*

| Sample | Raw_Reads | Clean_Reads | Clean_Rate% | Q20% | Q30% |
| --- | --- | --- | --- | --- | --- |
| CK_WX7_1 | 9985930 | 9568852 | 95.82 | 98.04 | 94.92 |
| CK_WX7_2 | 12273784 | 11557888 | 94.17 | 97.53 | 94.1 |
| CK_WX7_3 | 5769008 | 5471268 | 94.84 | 98.05 | 96.64 |
| CK_X4_1 | 11667140 | 11118490 | 95.3 | 97.84 | 94.52 |
| CK_X4_2 | 7292000 | 6904030 | 94.68 | 97.67 | 96.1 |
| CK_X4_3 | 13212776 | 12567040 | 95.11 | 97.74 | 94.37 |
| WX4_X_1 | 6475008 | 5984792 | 92.43 | 96.68 | 93.46 |
| WX4_X_2 | 11551268 | 11038872 | 95.56 | 97.94 | 94.31 |
| WX4_X_3 | 11671404 | 11187880 | 95.86 | 98.03 | 94.45 |
| WX7_X_1 | 10057811 | 9556932 | 95.02 | 97.78 | 94.23 |
| WX7_X_2 | 12497031 | 11952160 | 95.64 | 97.98 | 94.33 |
| WX7_X_3 | 11309383 | 10876234 | 96.17 | 98.28 | 95.59 |
| X4_X_1 | 11574212 | 10974668 | 94.82 | 97.66 | 94.11 |
| X4_X_2 | 11425513 | 10858808 | 95.04 | 97.74 | 94.33 |
| X4_X_3 | 11554733 | 10982774 | 95.05 | 97.71 | 94.17 |
| X7_X_1 | 12099314 | 11495558 | 95.01 | 97.7 | 94.24 |
| X7_X_2 | 10939848 | 10389574 | 94.97 | 97.69 | 94.17 |
| X7_X_3 | 12889117 | 12248528 | 95.03 | 97.77 | 94.41 |

Table S4 Sequencing Data Statistics of *C. capsici*

| Sample | Raw_Reads | Clean_Reads | Clean_Rate% | Q20% | Q30% |
| --- | --- | --- | --- | --- | --- |
| CK4_Z_1 | 47454586 | 44805438 | 94.42 | 95.56 | 93.67 |
| CK4_Z_2 | 48250456 | 45604878 | 94.52 | 95.56 | 93.64 |
| CK4_Z_3 | 52882506 | 50159432 | 94.85 | 95.77 | 93.9 |
| CK7_Z_1 | 32360526 | 30708364 | 94.89 | 95.57 | 93.68 |
| CK7_Z_2 | 38060882 | 35822966 | 94.12 | 95.52 | 93.59 |
| CK7_Z_3 | 41027300 | 38604684 | 94.1 | 95.59 | 93.67 |
| WX4_Z_1 | 37191798 | 35358868 | 95.07 | 95.64 | 93.72 |
| WX4_Z_2 | 48696648 | 45813826 | 94.08 | 95.38 | 93.44 |
| WX4_Z_3 | 44235226 | 42285652 | 95.59 | 95.83 | 93.66 |
| WX7_Z_1 | 49883112 | 46595650 | 93.41 | 95.16 | 93.08 |
| WX7_Z_2 | 32397490 | 30488544 | 94.11 | 95.38 | 93.41 |
| WX7_Z_3 | 48794876 | 46053900 | 94.38 | 95.44 | 93.51 |
| X4_Z_1 | 42703336 | 40616418 | 95.11 | 95.41 | 93.48 |
| X4_Z_2 | 45596914 | 43955144 | 96.4 | 96.6 | 94.66 |
| X4_Z_3 | 41203166 | 39763690 | 96.51 | 96.63 | 94.72 |
| X7_Z_1 | 37587326 | 36401096 | 96.84 | 96.85 | 95.01 |
| X7_Z_2 | 50523192 | 46998990 | 93.02 | 94.98 | 92.85 |
| X7_Z_3 | 46707892 | 43516682 | 93.17 | 94.93 | 92.79 |


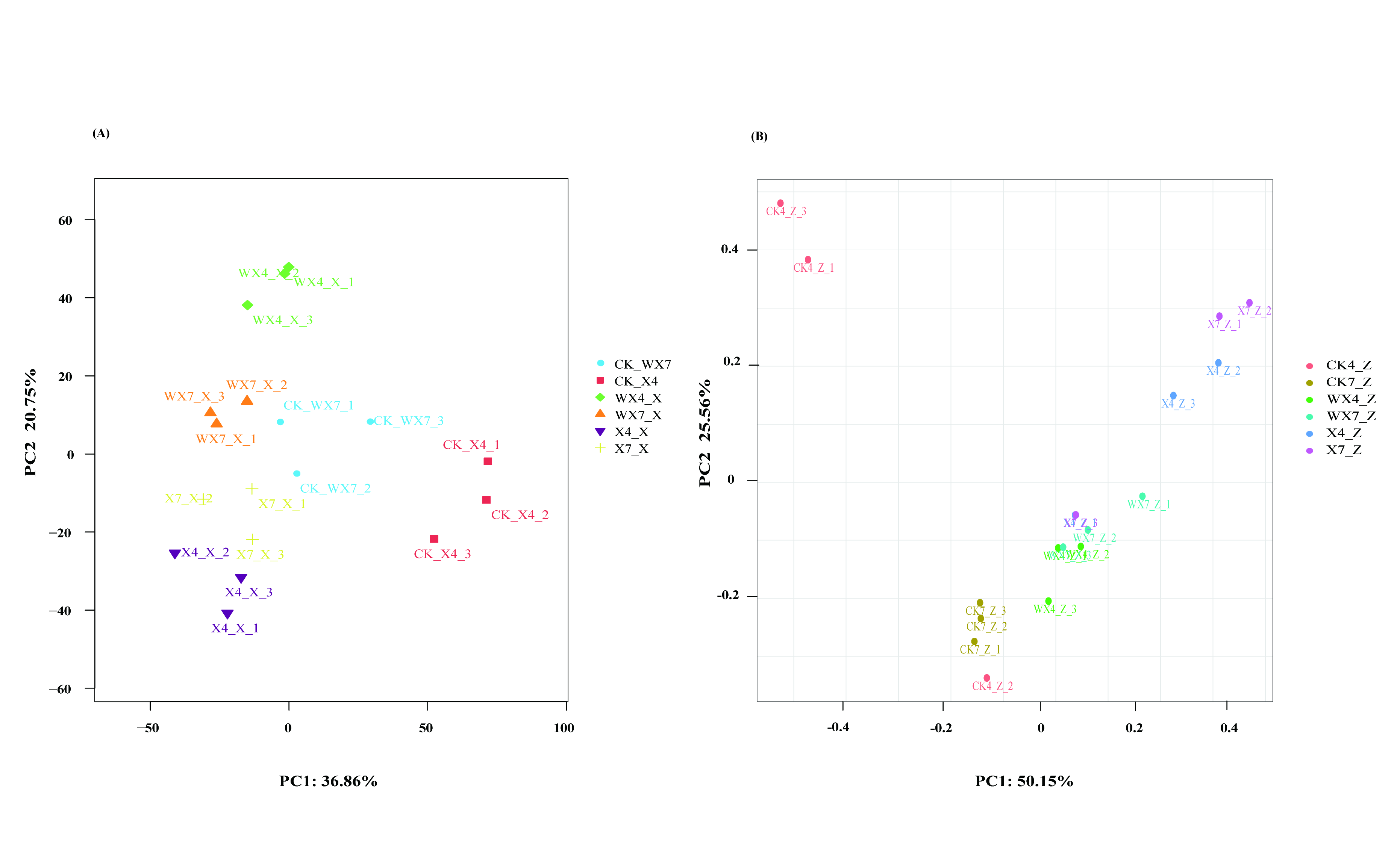


Figure S1 The PCA diagram of（A) *B. velezensis* and (B) *C. capsici*
